# Accumulation-depuration data collection in support of toxicokinetic modelling

**DOI:** 10.1038/s41597-022-01248-y

**Published:** 2022-03-30

**Authors:** Aude Ratier, Sandrine Charles

**Affiliations:** Université de Lyon, Université Lyon 1, CNRS UMR5558, Laboratoire de Biométrie et Biologie Evolutive, 69100 Villeurbanne, France

**Keywords:** Ecological modelling, Ecological modelling

## Abstract

Regulatory bodies require bioaccumulation evaluation of chemicals within organisms to better assess toxic risks. Toxicokinetic (TK) data are particularly useful in relating the chemical exposure to the accumulation and depuration processes happening within organisms. TK models are used to predict internal concentrations when experimental data are lacking or difficult to access, such as within target tissues. The bioaccumulative property of chemicals is quantified by metrics calculated from TK model parameters after fitting to data collected via bioaccumulation tests. In bioaccumulation tests, internal concentrations of chemicals are measured within organisms at regular time points during accumulation and depuration phases. The time course is captured by TK model parameters thus providing bioaccumulation metrics. But raw TK data remain difficult to access, most often provided within papers as plots. To increase availability of TK data, we developed an innovative database from data extracted in the scientific literature to support TK modelling. Freely available, our database can dynamically evolve thanks to any researcher interested in sharing data to be findable, accessible, interoperable and reusable.

## Abstract (summary)

**Table:** 

Measurement(s)	chemical concentration
Technology Type(s)	High-performance Liquid Chromatography-UV • radioactivity detection • high resolution mass spectrometry
Sample Characteristic - Organism	Gammarus pulex
Sample Characteristic - Environment	pond water
Sample Characteristic - Location	Canton of Zurich

## Background & Summary

The Environmental Risk Assessment (ERA) workflow for chemical substances of interest, as described in some European regulations (*e.g*., for plant protection products in marketing authorisation applications (EU regulation No 283/2013)), requires a bioaccumulation test, for example on fish according to the OECD Test guideline 305^1^. Such a test consists in an accumulation phase followed by a depuration one and the time course of the internal concentration within fish is measured at regular time points during both phases. The resulting data allow us to model the time-course of the exposure within organisms (denoted toxicokinetic, TK), summarized in the end via bioaccumulation metrics (BCF/BSAF/BMF for water, sediment and food exposure, respectively). From a regulatory point of view, these bioaccumulation metrics are key decision criteria to determine the bioaccumulative property of chemical substances^2^, and to further assess potential risks that are associated with them according to the exposure sources.

Kinetic bioaccumulation metrics are always defined as ratios between uptake and elimination rates, these latter being estimated from TK models^1,3^. More precisely, TK models relate the exposure concentration to a given chemical substance to the internal concentration within organisms, considering various processes such as absorption, depuration, metabolism and excretion (ADME)^4^. These last decades, different types of TK models have been proposed, all being compartment models^5,6^, where organs are considered as biological compartments connected through a fluid, usually blood. Organisms are thus described as whole or divided into organs, with input and output fluxes whose dynamics are described by TK models. Data collected from standard bioaccumulation tests are used to fit the TK models providing uptake and elimination rate estimates. They are also a way to provide a quantitative mechanistic framework to understand and simulate the time-course of the concentration of a chemical substance in various target organs accounting for body fluids in the case of physiologically-based toxicokinetic (PBTK) models. More generally, PBTK models allow to perform extrapolations that are inherent to risk assessment (*e.g*., extrapolations from one species to another, between exposure routes, from one exposure scenario to other ones, …), and to calculate bioaccumulation metrics^5^ thus helping decision-makers in the regulatory context.

Some years ago, the United States Environmental Protection Agency (US EPA) created a database for ecotoxicology data providing such bioaccumulation metrics according to species-compound combinations^7^. However, raw data are rarely provided in this database as this would require it to be collected directly from the corresponding scientific papers. Moreover, when available, raw data are mainly provided as plots. Nevertheless, to make updates or develop new TK modelling frameworks, it is crucial for researchers to be able to benefit from a collection of raw TK data in order to check the robustness of their new approaches. While developing the MOSAIC_*bioacc*_ web application^8^ (https://mosaic.univ-lyon1.fr/bioacc), we dealt with such a lack of raw data to fully test our innovative method. Indeed, MOSAIC_*bioacc*_ provides estimates of TK model parameters and bioaccumulation metrics with their uncertainties for a large range of species-compound combinations (*e.g*., aquatic or terrestrial organisms exposed to metals, hydrocarbons, active substances, etc.), encompassing different exposure routes and elimination processes. In particular, it was difficult to collect raw TK data with biotransformation processes being involved^9^, preventing us from fully testing the robustness of MOSAIC_*bioacc*_ for the widest possible diversity of input data types.

This motivated us in creating a new publicly available database as presented in this paper. This new database gathers together more than 200 datasets of published bioaccumulation tests, concerning more than 50 genus and more than 120 chemical substances. Some datasets concern several exposure routes (water, soil or sediment, and/or food) and several possible elimination processes (excretion, growth dilution and/or biotransformation). All the collected data were standardized in the same units and uploaded in MOSAIC_*bioacc*_, ensuring the use of the same methodology in the acces of bioaccumulation metrics for all the datasets. This database should allow the current lack of raw TK data in ecotoxicology to be overcome. Indeed, our purpose with this first version of the database is to motivate other researchers to share their data based on the Findability, Accessibility, Interoperability and Reuse (FAIR) principles which have become today almost a duty^10^. Our database can be considered as a proof of concept of the added-value of sharing raw TK data. It allows anyone to reuse data, for example to test new modelling frameworks. This database should also facilitate the design of new bioaccumulation experiments, as well as the comparison of results between several studies, benefiting from the unified calculation method for bioaccumulation metrics as provided by MOSAIC_*bioacc*_. Finally, we hope that this database could help in changing the paradigm in ecotoxicology, through an increase in a more wide sharing of raw data that would certainly lead to better knowledge in the perspective of ERA.

## Methods

**Fig. 1:**
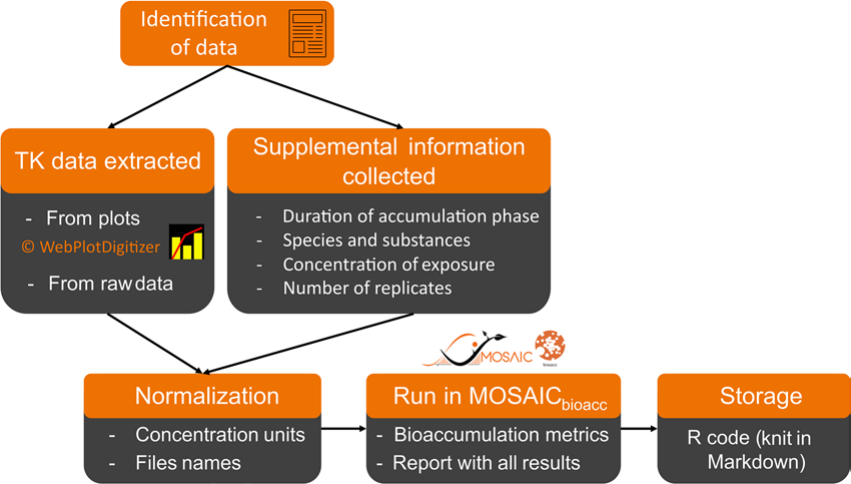
Conceptual framework of the collection of TK data and their storage in the database of MOSAIC_*bioacc*_.

The conceptual workflow we followed to collect the raw TK data is summarized on Fig. 1 and detailed below.

### Data and literature sources

One of our aims was to test the robustness of the MOSAIC_*bioacc*_ methodological framework generic feature from a wide collection of TK datasets, encompassing a variety of genus, chemical substances, exposure routes and elimination processes. For this purpose, the US EPA ecotoxicology database^7^ could not be used as raw observations are usually absent together with a lack of information (*e.g*., biotransformation processes are usually not informed). Consequently, an in-depth search in literature with Scopus was performed with specific keywords according to the expected type of TK datasets. These keywords were for example: “TK model aquatic”, “TK model terrestrial”, “TK model biotransformation”, “TK biotransformation rate”, “TK model food exposure”, “TK model sediment exposure”, “TK model water exposure”, etc (Table S1). For each retained article, we manually checked if TK data were available in tables or plots directly, in the manuscript or in the supporting information. A total of 56 studies were finally selected, fully analysed with a TK model and gathered in our database as a proof-of-concept. So, our corpus of data is therefore anything but exhaustive of what is available in the scientific literature.

### Toxicokinetic data

#### Literature source data extraction

Only few publications provided raw data as directly usable tables of internal concentrations of a chemical (and its potential metabolite(s)) within organisms versus time. For most of the studies, data were only available in plots as dots to digitize. Plots were converted into JPEG files from screenshots (native screenshot tool in Windows 10). Then images were imported using WebPlotDigitizer^11^ to extract the underlying numerical values corresponding to dots on plots. The calibration of axes and the selection of dots for extraction were performed manually. Finally, raw data are exported from WebPlotDigitizer to comma-separated values (CSV) files. Those CSV files of data were formatted following MOSAIC_*bioacc*_ requirements, manually adding information on replicates and exposure concentration according to the Methods section of each article. For each collected TK dataset, the corresponding file was named by the genus, the chemical substance, the duration of the accumulation phase, the first author and the year of publication, possibly adding comments (*e.g*., number of tested organism) if available.

#### Data standardization

Measured data were standardized to always express exposure concentrations in *μg.ml*^−1^ (for water exposures) or in *μg.g*^−1^ (for sediment and food exposures). The internal chemical concentrations within organisms were translated into *μg.g*^−1^, as required for a proper use of MOSAIC_*bioacc*_.

#### TK modelling and inference framework under MOSAIC_*bioacc*_

MOSAIC_*bioacc*_ fits a one-compartment TK model written as a set of four ordinary differential equations (two per phases) to observed data. The inference process is implemented under a Bayesian framework based on a Monte-Carlo Markov Chain (MCMC, three chains by default) algorithm. The full model (and its generic solving, whatever the number of exposure routes and the number of phase I metabolites), as well as the inference method specifically adapted to get all parameter estimates simultaneously, are fully detailed in^12^ and^13^, respectively. New insights on the calculation of bioaccumulation metrics are provided in^8,14^. The user guide of MOSAIC_*bioacc*_ that is available on-line from http://lbbe-shiny.univ-lyon1.fr/mosaic-bioacc/data/user_guide.pdf also explains the whole modelling process, together with details on the minimal data requirements that are expected to use MOSAIC_*bioacc*_: at least one exposure concentration value, and time points with the corresponding measured internal concentrations; only accumulation data (during the accumulation phase of the test) are mandatory.

#### MOSAIC_*bioacc*_ running

Once standardized, each collected dataset was uploaded into MOSAIC_*bioacc*_ which automatically provided both kinetic and steady-state bioaccumulation metrics, unifying their calculation among datasets allowing relevant comparisons or a correct classification of chemical substances according to their bioaccumulative capacity for example. Kinetic bioaccumulation metrics (summarized as medians and 95% uncertainty intervals) were saved in the dedicated repository, and the report with all fitting outputs downloaded in the same repository, for each dataset. All calculations performed with MOSAIC_*bioacc*_ can also be run directly in the R software^15^ with the new rbioacc package^16^.

## Data Records

### Storage and display

All collected datasets (directly downloadable as tabular files), the bibtex file with all references, all reports and all kinetic bioaccumulation metric estimates are publicly available on Zenodo^17^. An rmarkdown file^18,19^ was created to build the overview table with information collected from the name of the dataset and from the dataset itself (*e.g*., column headers, number of data, number of replicates), as well as from the bibtex file. The R package DT was additionally used^20^ to combine all collected information in a user-friendly manner including a convenient search tool, and the rmarkdown file was finally compiled^19^ in HTML format for display to the user in packs of 10 lines by default. In such a way, each new dataset added into the repository will compile the rmarkdown file automatically for update.

**Fig. 2:**
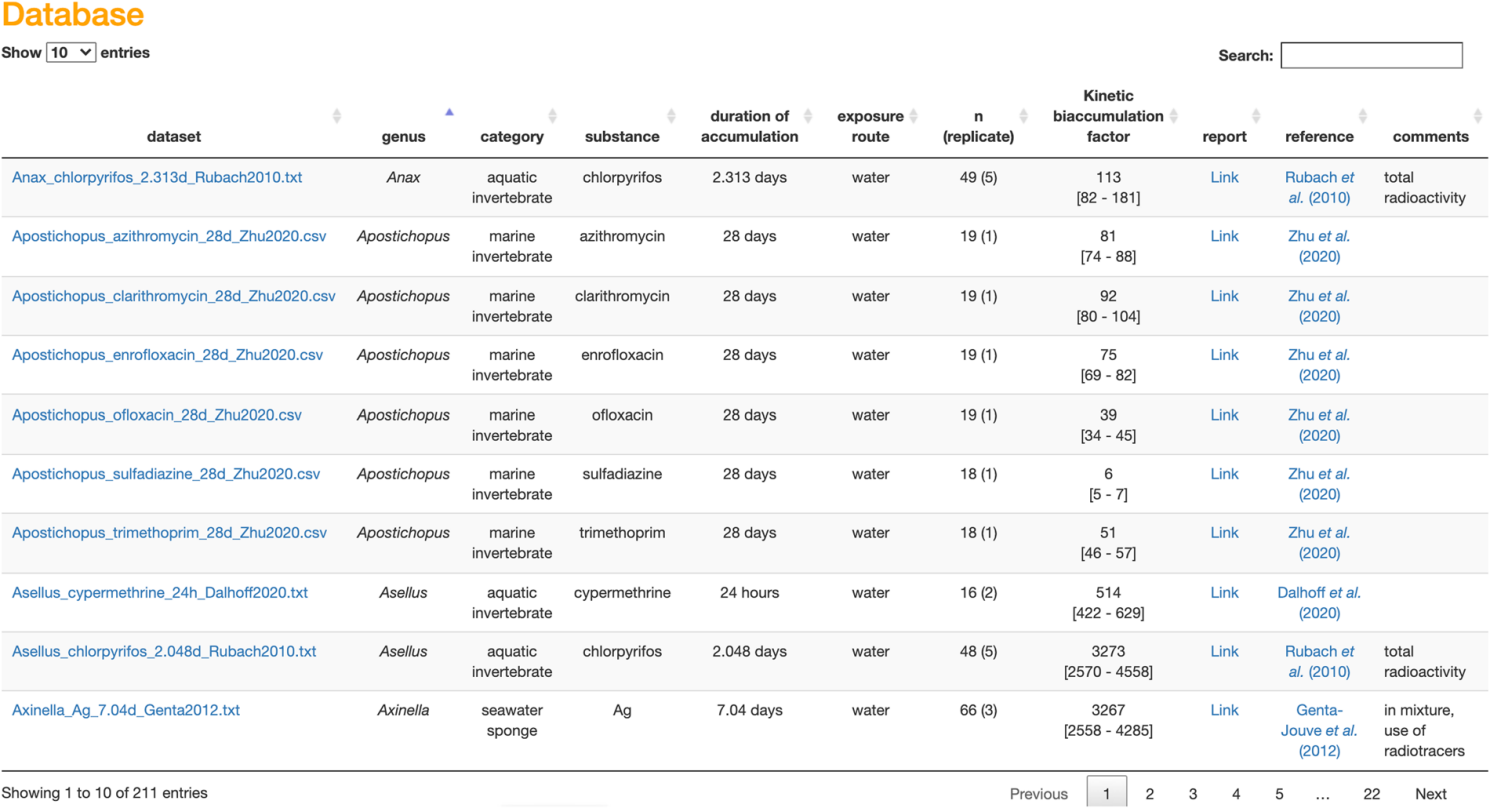
Screenshot of the first page of the overview table of the database available from MOSAIC_*bioacc*_.

In parallel, the database can also be accessed directly via http://lbbe-shiny.univ-lyon1.fr/mosaic-bioacc/data/database/TK_database.html, or from MOSAIC_*bioacc*_ clicking on the “More scientific TK data” button. An example of the output of the overview table is shown in Fig. 2, while the full table is provided in the supplementary information (Table S2). The collected raw TK data of the database consist in the time-course of several types of chemical substances bioaccumulated in various species via different exposure routes.

### Datasets overview

Each dataset is summarized by:

- the file name (raw data directly downloadable by clicking on the file name, in text or CSV format),
- the genus of the tested organism,
- the category of the organism (*e.g*., aquatic, terrestrial, etc.),
- the tested chemical substance,
- the duration of the accumulation phase,
- the tested exposure routes (*e.g*., water, sediment, food, pore water),
- the total number of observations in the dataset (plus the number of replicate(s) in brackets),
- the kinetic bioaccumulation metric median value with its 95% uncertainty interval,
- the report which contains all the outputs from MOSAIC_*bioacc*_ (in PDF format),
- the link to the reference or the source of the data,
- some additional comments (*e.g*., lipid fraction, growth, biotransformation, if exposure was done for chemical mixtures or not, if total radioactivity was used or not, etc.).

A summary of all datasets is presented in Table 1. Genus were separated in 12 categories: aquatic invertebrates (*n* = 105), fish (*n* = 42), insects (*n* = 17), aquatic worms (*n* = 10), terrestrial worms (*n* = 16), seawater sponges (*n* = 2), seawater plants (*n* = 1), aquatic algae (*n* = 1), terrestrial invertebrates (*n* = 1), vertebrates other than fish (*n* = 4), marine invertebrates (*n* = 8), and heterotrichea (*n* = 4). The most represented genus in the database are *Gammarus* (aquatic invertebrate, *n* = 43) and *Daphnia* (aquatic invertebrate, *n* = 27), followed by *Oncorhynchus* (fish, *n* = 15), genus that are classically used in ecotoxicological experiments. Recommended genus by OECD guidelines for bioaccumulation tests are *Eisenia* and *Enchytraeus* for terrestrial organisms (OECD 317)^21^, and *Tubifex* or *Lumbriculus* for aquatic invertebrates exposed to sediment (OECD 315)^22^; some datasets for these specific species are available in the database (*n* = 24).

**Table 1:** Summary of the collected TK datasets.

Genus (*n* = 52)	chemical substances (*n* = 124)	Exposure route	Elimination process	Kinetic bioaccumulation metric (median)	Number of studies
freshwater invertebrate (*n* = 105)	metals (*n* = 20)	water (*n* = 137)	biotransformation (*n* = 34)	BCF < 1000 (*n* = 88)	56
fish (*n* = 42)	PCB (*n* = 22)	food (*n* = 38)	excretion (*n* = 211)	1000 < BCF < 2000 (*n* = 9)	
insect (*n* = 17)	pesticides (*n* = 71)	sediment (*n* = 36)		2000 < BCF < 5000 (*n* = 15)	
aquatic worm (*n* = 10)	flame retardants (*n* = 8)			BCF > 5000 (*n* = 25)	
terrestrial worm (*n* = 16)	pharmaceutical products (*n* = 14)			BSAF > 1 (*n* = 16)	
seawater sponge (*n* = 2)	hydrocarbons (*n* = 37)			BSAF < 1 (*n* = 20)	
seawater plant (*n* = 1)	octyphenols (*n* = 2)			BMF > 1 (*n* = 8)	
aquatic algae (*n* = 1)	PFAS (*n* = 7)			BMF < 1 (*n* = 30)	
terrestrial invertebrate (*n* = 1)	nanoparticles (*n* = 23)				
vertebrate (other than fish) (*n* = 4)	other (*n* = 7)				
marine invertebrate (*n* = 8)					
heterotrichea (*n* = 4)					

The classification of bioaccumulative properties are the same as in ECHA (2017)^2,25^.

Chemical substances were divided in 10 classes following at the best the nomenclature used in Standartox^23^: pesticides (*n* = 71), hydrocarbons (*n* = 37), metals (*n* = 20), nanoparticules (*n* = 23), polychlorobiphenyls (PCB, *n* = 22), flame retardants (brominated or chlorinated, *n* = 8), pharmaceutical products (*n* = 14), PFAS (*n* = 7), octyphenol (*n* = 2) and other (*n* = 7). Among all datasets, the majority of bioaccumulation tests were performed via spiked water (*n* = 137). Besides, 34 datasets account for biotransformation processes, considering from 1 to 8 metabolites.

According to ECHA (2017)^2^, BCF below 1,000 means that the chemical substance is not bioaccumulative, whereas one ranging between 1,000 and 5,000 corresponds to a bioaccumulative chemical substance: low bioaccumulative if BCF ∈]1,000; 2,000]; mid-bioaccumulative if BCF ∈]2,000; 5,000]. If BCF is >5000, the chemical substance is classified as very bioaccumulative. These ranges are reported in Table 1, where BCF median estimates are >5000 for 25 datasets, indicating a very bioaccumulative capacity of the corresponding chemical substances for the corresponding genus. Concerning BSAF and BMF estimates, their value must be compared to threshold 1. A median BSAF estimate >1 indicates that the corresponding chemical substance can bioaccumulate from soil or sediment into organisms at the base of the non-aquatic food chain^24,25^; a median BMF estimate >1 indicates that the corresponding chemical substance can biomagnify in the trophic relationship under consideration^26^. In the database, 16 datasets in 36 led to BSAF >1, for genus *Eisenia* (n = 2), *Enchytraeus* (n = 6), *Gallus* (n = 1), *Lumbriculus* (n = 2), *Metaphire* (n = 2), *Physa* (n = 1), *Radix* (n = 2)), while 8 datasets in 38 led to BMF >1, for genus *Gallus* (n = 1), *Oncorhynchus* (n = 5) and *Perca* (n = 2). On an ecotoxicological point of view, the highest BCF estimates were obtained for genus *Culex* and *Sialis* exposed to chlorpyrifos due to a very low estimate of the elimination rate, for genus *Gammarus* and *Calanus* exposed to hydrocarbons, and several aquatic invertebrates exposed to pesticides, especially chlorpyrifos (*n* = 4), attesting to the potential high bioaccumulation capacity and high risk of toxicity associated with this chemical substance for aquatic organisms. Overall, aquatic invertebrates seem to be the most sensitive category of organisms in terms of bioaccumulation of chemical substances representing 20 in the 25 datasets with a BCF estimates >5000.

## Technical Validation

The validity of the collected data was checked by considering several aspects in the corresponding papers: (i) limitations in the material and method section, such as for example unreported numbers of replicates, avoiding to account for the variability between replicates; (ii) not enough details regarding the experimental design preventing reproducibility of the measurements (*e.g*., only chemical name (source of ambiguity), the frequency of the renewal of the exposure media, etc.); (iii) the quality of the fitting results gotten from MOSAIC_*bioacc*_ when reanalyzed, that is goodness-of-fit (GOF) criteria that were not satisfying enough, or low precision of parameter estimates.

For some studies, raw data were available in the supplementary files or upon request from the authors themselves, but appeared sometimes not consistent with the associated plots as provided within the original paper. For example, we sometimes detected some discrepancies in the plots, what led us to remove the corresponding datasets from our database. Besides, most of the studies did not provide the real raw data at all, such as separated concentration measurements in each replicate. Hence, most of the time, plots only provided average measurements with error bars standing for standard deviation, standard error or other uncertainty measures, thus preventing a full re-use of the data which are then reduced to mean values only.

The uncertainty due to data extraction from plots with the WebPlotDigitizer tool^11^ is legitimate to account for as a bias may be introduced when digitizing dots. To quantify this bias, we performed the digitization of TK data from dots of a same plot (first saved as a .jpeg file) ten times and calculated the extraction errors possibly due to the shape of points, the axes calibration, the image resolution or other error sources. Hence, a variation of 0.12 ± 0.13% was quantified for the dataset of Chen *et al*.^27^ (Fig. 2a in their paper), which can be considered as relatively low, given that values less than 0.5% are usually accepted for coefficients of variation. This result was representative of what we had for most of the plots, reinforcing our approach to digitizing the raw data.

**Fig. 3:**
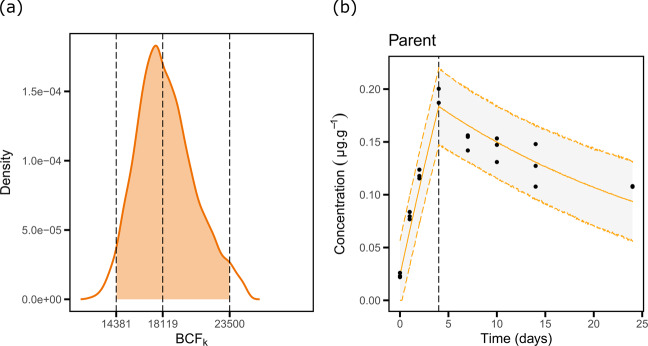
Example of results given by MOSAIC_*bioacc*_: (**a**) the bioaccumulation metric and (**b**) the fitted TK model predictions with observed data. This example comes from the dataset ‘Male_Gammarus_Single.txt’ available from MOSAIC_*bioacc*_, where male gammarids were exposed to mercury (Hg) for 4 days in spiked water.

In order to further promote the quality of the data in our database, MOSAIC_*bioacc*_ was used to fit the appropriate TK models to all datasets, thus benefiting from the same conceptual framework and then make relevant comparisons. Indeed, bioaccumulation metrics and TK model fitting outputs were systematically provided (see an example on Fig. 3), as well as a collection of identical GOF criteria allowing for *a posteriori* check of the results. All this information was finally gathered together in a PDF report downloadable from the MOSAIC_*bioacc*_ database for each dataset.

More than 95% of the datasets in the MOSAIC_*bioacc*_ database were already published, meaning that they were publicly available for re-use. The re-analysis with MOSAIC_*bioacc*_ allowed us to compare fitting results to those of the original study and to confirm the relevance of including the corresponding data in the database. In addition, very few of the publications (7 among 56) provided uncertainty around the predicted concentrations (at least, not visualized in the original plots), while we systematically associated the 95% credible intervals around median predicted estimates, as well as uncertainties for all bioaccumulation metrics, for each dataset. We are thus in full compliance with the recent recommendations from EFSA^28^.

## Usage Notes

Raw TK data are particularly useful to better understand the relationship between exposure chemical concentrations in the environment and their potential bioaccumulation within living organisms. They are also beneficial to researchers or regulators when evaluating the bioaccumulative capacity of chemical substances that is directly linked to potential toxic effects on life-history traits of the organisms. Our database also facilitates the access to tabular raw TK data that can be used to fit current TK models (with one or several compartment(s)), or to develop new conceptual modelling frameworks. It can also serve to simulate the time-course of the concentration of a chemical substance in various organs and bodily fluids in the case of PBTK models. Given that recommendations become more and more restrictive regarding the Replacement, Reduction and Refinement (3 R) principles^29^, our database could reveal itself useful to perform extrapolations from one species, one exposure or one exposure scenario to any other ones of interest, in a change of paradigm in terms of model-guided experiments.

Our database does not represent an exhaustive overview of all available TK data in literature, but has the distinct advantage of encompassing several types of genus, chemical substances, exposure routes and elimination processes, thus constituting a supportive proof-of-concept of the feasibility and the major interest in sharing research data. Our database also aimed at developing and validating our generic TK modelling framework, as illustrated in this paper where the evaluation of the efficiency and robustness of MOSAIC_*bioacc*_ proved to be successful for the whole set of collected data^9^. This is very much in line with Open Science as advocated today by most of research bodies^30^.

When bioaccumulation testing is required by regulatory documents, it may be difficult to appropriately plan the experimental design which depends on the chemical substance, the species and the exposure route under consideration. Usually, a search in literature provides a possible range of exposure concentrations for the chemical substance, but the amount likely to be bioaccumulated in organisms of the chosen species remains unknown, as well as how long it will require to reach the steady-state at the end of the accumulation phase. In this perspective, the MOSAIC_*bioacc*_ database quick search option (Fig. 2) may help to look at data on phylogenetically close genus and/or chemical substances with similar modes of action to drive new experiments.

Finally, our database could support further research to increase our knowledge on bioaccumulation of chemical substances for which there are clear societal challenges in terms of environmental protection today. In terms of regulatory ERA, bioaccumulation metrics are key decision criteria that classify a chemical substance as non-bioaccumulative, bioaccumulative or very bioaccumulative. Most often, these metrics are calculated from two or three different modelling approaches, which makes their comparison difficult. In addition, they are usually provided only as point mean values without uncertainty. Our database, together with the use of MOSAIC_*bioacc*_, could contribute in improving this classification thanks to kinetic bioaccumulation metrics estimated with the same methodology for all datasets, with the quantification of the uncertainty summarized by the 95% credible intervals around median values. More globally, our database could encourage researchers to share their data with the scientific community by directly providing the raw data in their publications, as required by the FAIR data principles.

## Supplementary information

Supplementary Information

## Data Availability

In order to reproduce all the outputs provided in the PDF reports for all the datasets of the database: once downloaded, raw data can be uploaded in MOSAIC_*bioacc*_ to perform the statistical analysis on-line. In addition, for each dataset, the corresponding R code can be retrieved from the download section of MOSAIC_*bioacc*_, if using the R software is preferred.
