# Supplementary material for: Accumulation-depuration data collection in support of toxicokinetic modelling
Source: Sci Data. 2022 Mar 30;9:130. doi: 10.1038/s41597-022-01248-y (PMC8967850; doi:10.1038/s41597-022-01248-y)
Supplement: Supplementary file 1 — Supplementary Information [file 41597_2022_1248_MOESM1_ESM.pdf]

Supplementary Information: Accumulation-depuration data collection in support of toxicokinetic modelling

Aude Ratier[1] **and Sandrine Charles**[1,*]

[1]Université de Lyon, Université Lyon 1, CNRS UMR5558,
Laboratoire de Biométrie et Biologie Evolutive, 69100 Villeurbanne, France
[*]corresponding author: Sandrine Charles (sandrine.charles@univ-lyon1.fr)

Keywords

In Table S1 below are presented the results of the literature search in Scopus. The number of retrieved publications appear in a chronological order, *i.e.*, there is no additional count if the study was already found with the previous keywords.

Table S1. Keywords used for the literature bibliography with the associated number of publications that were retrieved accordingly.

Utilized keywords	Total number of publications	Retained publications
`TK model aquatic`	161	7
`Toxicokinetic freshwater aquatic`	32	4
`TK model terrestrial`	149	3
`TK model biotransformation`	26	4
`TK biotransformation rate`	9	2
`TK model food exposure`	98	1
`TK model dietary exposure`	29	1
`TK model sediment exposure`	29	2
`TK model water exposure`	136	3
`bioaccumulation soil toxicokinetic`	30	3
`earthworm toxicokinetic`	38	4
`bioaccumulation sediment`	6,522 (only the first 100 were checked)	1
`bioaccumulation earthworm biotransformation`	40	1
`bioaccumulation food exposure`	2,752 (only the first 100 were checked)	2
`bioaccumulation biotransformation aquatic`	366 (only the first 100 were checked)	3
`bioaccumulation kinetic`	602 (only the first 100 were checked)	15

Overview of the database

Table S2 below shows the whole database as displayed directly on-line from
http://lbbe-shiny.univ-lyon1.fr/mosaic-bioacc/data/database/TK_database.html
Note that all raw data can be downloaded from
https://zenodo.org/badge/latestdoi/359723940

Table S2. Accumulation-depuration data collection in support of toxicokinetic modelling.

genus	category	substance	tacc	tunit	exposure	ndata	nrep	bioacc	bioaccQinf	bioaccQsup	DOI	comment
Asellus	aquatic invertebrate	cypermethrine	24	hours	water	16	2	514	422	629	http://dx.doi.org/10.1021/acs.est.0c00189	
Axinella	seawater sponge	Ag	7.04	days	water	66	3	3267	2558	4285	http://dx.doi.org/10.1016/j.chemosphere.2012.04.052	in mixture, use of radio-tracers
Axinella	seawater sponge	Hg	7.04	days	water	66	3	3275	2561	4271	http://dx.doi.org/10.1016/j.chemosphere.2012.04.052	in mixture, use of radio-tracers
Calanus	aquatic invertebrate	dodecane	88	hours	water	17	3	118756	33521	2509635	http://dx.doi.org/10.1016/j.aquatox.2017.12.001	use of radiotracers
Calanus	aquatic invertebrate	phenanthrene	88	hours	water	16	3	117733	82830	202192	http://dx.doi.org/10.1016/j.aquatox.2017.12.001	use of radiotracers
Chaetopteryx	insect	cypermethrine	24	hours	water	16	2	40	32	49	http://dx.doi.org/10.1021/acs.est.0c00189	
Chironomus	aquatic invertebrate	cypermethrine	24	hours	water	16	2	209	173	252	http://dx.doi.org/10.1021/acs.est.0c00189	
Chlorella	aquatic algae	4-tert-octylphenol	24	hours	water	11	2	531	0	1165	http://dx.doi.org/10.1016/j.ecoenv.2020.110379	
Danio	fish	atrazine	48	hours	water	30	3	11	10	11	https://doi.org/10.1016/j.scitotenv.2012.02.065	2 tested concentrations
Danio	fish	chlorpyrifos	48	hours	water	35	6	1247	1097	1398	https://doi.org/10.1016/j.scitotenv.2012.02.065	2 tested concentrations
Danio	fish	dicofol	48	hours	water	33	3	5153	4707	5682	https://doi.org/10.1016/j.scitotenv.2012.02.065	
Daphnia	aquatic invertebrate	4-tert-octylphenol	9	hours	water	14	2	3188	2243	4454	http://dx.doi.org/10.1016/j.ecoenv.2020.110379	
Daphnia	aquatic invertebrate	alpha-cypermethrine propi-conazole	7.5	hours	water	24	3	511	351	733	https://doi.org/10.1007/s00216-015-9197-x	
Daphnia	aquatic invertebrate	alpha-cypermethrine	7.5	hours	water	22	2	364	302	427	https://doi.org/10.1007/s00216-015-9197-x	
Daphnia	aquatic invertebrate	cypermethrine	24	hours	water	16	2	562	472	650	http://dx.doi.org/10.1021/acs.est.0c00189	
Daphnia	aquatic invertebrate	diazinon	21	hours	water	30	6	428	399	457	https://doi.org/10.1021/es104324v	
Daphnia	aquatic invertebrate	imidacloprid	90	hours	water	36	3	1183	1150	1215	https://doi.org/10.1021/acs.est.0c05664	
Daphnia	aquatic invertebrate	TPT	72	hours	water	9	1	793	0	1635	https://doi.org/10.1021/acs.est.6b01113	intermediate size, in mixture
Daphnia	aquatic invertebrate	TPT	72	hours	water	8	1	1023	0	27280	https://doi.org/10.1021/acs.est.6b01113	large size, in mixture
Daphnia	aquatic invertebrate	TPT	72	hours	water	9	1	1406	671	2143	https://doi.org/10.1021/acs.est.6b01113	small size, in mixture
Eisenia	aquatic worm	tebuconazol	21	hours	sediment	24	3	3	2	3	https://doi.org/10.1016/j.envpol.2018.01.082	in mixture
Enchytraeus	terrestrial worm	Ce	14	days	water	6	6	4	0	59518	https://www.sciencedirect.com/science/article/pii/S0269749119373673	6 tested concentrations
Enchytraeus	terrestrial worm	Gd	14	days	water	6	6	0	0	1465	https://www.sciencedirect.com/science/article/pii/S0269749119373673	6 tested concentrations
Enchytraeus	terrestrial worm	La	14	days	water	6	6	0	0	919	https://www.sciencedirect.com/science/article/pii/S0269749119373673	6 tested concentrations
Ephemera	insect	cypermethrine	24	hours	water	16	2	38	24	53	http://dx.doi.org/10.1021/acs.est.0c00189	
Gammarus	aquatic invertebrate	2,4-dichloro-aniline	0.999	days	water	14	2	8	0	6229	http://dx.doi.org/10.1021/es204611h	biotransformation, total radioactivity
Gammarus	aquatic invertebrate	2,4,5-trichlorophenol	0.979	days	water	18	2	43	0	47094	http://dx.doi.org/10.1021/es204611h	biotransformation, total radioactivity
Gammarus	aquatic invertebrate	4-nitrobenzyl-chloride	1	days	water	14	2	223	6	806025	http://dx.doi.org/10.1021/es204611h	biotransformation, total radioactivity
Gammarus	aquatic invertebrate	aldicarb	1	days	water	14	2	7	4	18	http://dx.doi.org/10.1021/es204611h	biotransformation, total radioactivity
Gammarus	aquatic invertebrate	azoxistrobine	1	days	water	38	2	3982	1666	7674598	https://doi.org/10.1021/acs.est.6b01301	biotransformation
Gammarus	aquatic invertebrate	BDE99	7	days	food	8	1	0.0473	0.0335	0.0635	https://tel.archives-ouvertes.fr/tel-02612792	
Gammarus	aquatic invertebrate	BDE99	7	days	sediment	8	1	0.1629	0.1233	0.2138	https://tel.archives-ouvertes.fr/tel-02612792	
Gammarus	aquatic invertebrate	carbaryl	0.990	days	water	20	2	5	3	11	http://dx.doi.org/10.1021/es204611h	biotransformation, total radioactivity
Gammarus	aquatic invertebrate	carbofuran	0.990	days	water	18	2	1	0	585	http://dx.doi.org/10.1021/es204611h	biotransformation, total radioactivity
Gammarus	aquatic invertebrate	chlorpyrifos	0.979	days	water	15	2	132	76	184	http://dx.doi.org/10.1021/es204611h	biotransformation, total radioactivity
Gammarus	aquatic invertebrate	Cu	7	days	water	92	15	10116	9286	11029	https://www.sciencedirect.com/science/article/pii/S0166445X1730303X	
Gammarus	aquatic invertebrate	cypermethrine	24	hours	water	16	2	202	128	283	http://dx.doi.org/10.1021/acs.est.0c00189	
Gammarus	aquatic invertebrate	diazepam	2	days	water	12	3	151	36	182457	https://www.sciencedirect.com/science/article/pii/S0045653517307877	biotransformation
Gammarus	aquatic invertebrate	dichlorophenol	0.979	days	water	20	2	2	0	1866	http://dx.doi.org/10.1021/es204611h	biotransformation, total radioactivity
Gammarus	aquatic invertebrate	HBCD	9	days	sediment	17	2	0.0363	0.022	0.0499	https://tel.archives-ouvertes.fr/tel-02612792	commercial mixture
Gammarus	aquatic invertebrate	malathion	0.99	days	water	18	2	7660	22	818346	http://dx.doi.org/10.1021/es204611h	biotransformation, total radioactivity
Gammarus	aquatic invertebrate	Ni	7	days	water	87	14	276	257	294	https://www.sciencedirect.com/science/article/pii/S0166445X1730303X	
Gammarus	aquatic invertebrate	PCB153	7	days	food	11	3	0.0702	0.0602	0.0817	https://tel.archives-ouvertes.fr/tel-02612792	
Gammarus	aquatic invertebrate	PCB153	7	days	sediment	9	2	0.4038	0.2502	0.7573	https://tel.archives-ouvertes.fr/tel-02612792	
Gammarus	aquatic invertebrate	PeCB	6	days	food	11	1	0.0952	0.0754	0.1168	https://tel.archives-ouvertes.fr/tel-02612792	

Go to next page

Table S2. *(continued)*.

genus	category	substance	tacc	tunit	exposure	ndata	nrep	bioacc	bioaccQinf	bioaccQsup	DOI	comment
Gammarus	aquatic invertebrate	pentachlorophenol	0.990	days	water	20	2	105231	894	6452195	http://dx.doi.org/10.1021/es204611h	biotransformation, total radioactivity
Gammarus	aquatic invertebrate	propranolol	2	days	water	12	3	27	18	35	https://www.sciencedirect.com/science/article/pii/S0045653517307877	biotransformation
Gammarus	aquatic invertebrate	PRZ	1	days	water	38	2	132	122	143	https://doi.org/10.1021/acs.est.8b04057	biotransformation, in mixture
Gammarus	aquatic invertebrate	pyrene	7	days	water	28	4	145030	1755	8144775	https://www.sciencedirect.com/science/article/pii/S0141113615300113	biotransformation, total radioactivity
Halophila	seawater plant	Ni	12	days	water	6	1	1	0	55	http://dx.doi.org/10.1016/j.ecoenv.2020.111386	total radioactivity
Heptagenia	insect	cypermethrine	24	hours	water	16	2	1414	632	54717	http://dx.doi.org/10.1021/acs.est.0c00189	
Hyalella	aquatic invertebrate	AZ	1	days	water	38	2	15	12	21	https://doi.org/10.1021/acs.est.8b04057	biotransformation, in mixture
Hyalella	aquatic invertebrate	BaP	3	days	sediment	14	1	0.315	0.27	0.365	http://dx.doi.org/10.1002/etc.5620220227	total radioactive residue
Hydropsyche	insect	cypermethrine	24	hours	water	16	2	541	83	29813	http://dx.doi.org/10.1021/acs.est.0c00189	
Lumbriculus	aquatic worm	BaP	10.5	days	sediment	29	3	1	1	1	http://dx.doi.org/10.1002/etc.5620220227	total radioactive residue
Lumbriculus	aquatic worm	cd	14	days	sediment	8	2	0.3497	0.1789	0.6237	http://dx.doi.org/10.1007/s10646-017-1769-4	
Lumbriculus	aquatic worm	fipronil	14	days	sediment	9	1	0.093	0.0767	0.1093	http://dx.doi.org/10.1016/j.scitotenv.2019.03.490	
Oncorhynchus	fish	2-6dimethyldecane	14	days	food	27	3	3.5e-03	2.6e-03	5e-03	https://doi.org/10.1002/etc.3050	
Oncorhynchus	fish	BaPy	14	days	food	29	3	0.0452	0.0231	0.0876	https://doi.org/10.1002/etc.3050	
Oncorhynchus	fish	chrysene	14	days	food	25	4	0.0124	0.0102	0.0148	https://doi.org/10.1002/etc.3050	
Oncorhynchus	fish	hexachlorobenzene	14	days	food	29	5	2	1	17	https://doi.org/10.1002/etc.3050	
Oncorhynchus	fish	hexachlorocyclohexane	14	days	food	25	5	0	0	4	https://doi.org/10.1002/etc.3050	
Oncorhynchus	fish	hexylcyclohexane	14	days	food	32	5	0.0151	7.9e-03	0.0347	https://doi.org/10.1002/etc.3050	
Oncorhynchus	fish	methylanthracene	14	days	food	24	4	5.1e-03	4.2e-03	6.6e-03	https://doi.org/10.1002/etc.3050	
Oncorhynchus	fish	pcb153	14	days	food	34	5	6	1	87	https://doi.org/10.1002/etc.3050	
Oncorhynchus	fish	pcb155	14	days	food	32	5	8	1	109	https://doi.org/10.1002/etc.3050	
Oncorhynchus	fish	pcb209	14	days	food	34	5	9	1	117	https://doi.org/10.1002/etc.3050	
Oncorhynchus	fish	pcb52	14	days	food	31	5	4	1	63	https://doi.org/10.1002/etc.3050	
Oncorhynchus	fish	pentachlorobenzene	14	days	food	30	4	0.2104	0.1213	0.4352	https://doi.org/10.1002/etc.3050	
Oncorhynchus	fish	tetrachlorobenzene-benzene	14	days	food	25	4	0.154	0.0843	0.4202	https://doi.org/10.1002/etc.3050	
Oncorhynchus	fish	tetramethyl-benzene	14	days	food	24	4	7e-03	5.8e-03	9e-03	https://doi.org/10.1002/etc.3050	
Oncorhynchus	fish	trans-decalin	14	days	food	32	5	0.0148	0.0103	0.0216	https://doi.org/10.1002/etc.3050	
Oryzias	fish	PE	14	days	water	12	1	0.1489	0.1062	0.1868	http://dx.doi.org/10.1016/j.ecoenv.2021.112007	
Physa	aquatic invertebrate	3-8nmAgNP	7	days	water	21	3	70	6	4054	http://dx.doi.org/10.1039/d0en00946f	
Physa	aquatic invertebrate	50nmAgNP	7	days	water	18	3	42	2	2152	http://dx.doi.org/10.1039/d0en00946f	
Physa	aquatic invertebrate	60nmAgNP	7	days	water	22	3	129	10	7552	http://dx.doi.org/10.1039/d0en00946f	
Physa	aquatic invertebrate	Ag2S-nP	7	days	water	23	4	5	3	7	http://dx.doi.org/10.1039/d0en00946f	
Physa	aquatic invertebrate	AGNO3	7	days	sediment	24	3	25262	0	1706990	http://dx.doi.org/10.1039/d0en00946f	
Planorbarius	aquatic invertebrate	azinphos-methyl	12	hours	water	27	4	13	12	13	https://www.sciencedirect.com/science/article/pii/S0166445X1830345X	
Planorbarius	aquatic invertebrate	AZM in mixture with carbaryl	12	hours	water	28	4	12	11	13	https://www.sciencedirect.com/science/article/pii/S0166445X1830345X	in mixture
Planorbarius	aquatic invertebrate	carbaryl in mixture with AZM	12	hours	water	27	4	4	4	4	https://www.sciencedirect.com/science/article/pii/S0166445X1830345X	in mixture
Planorbarius	aquatic invertebrate	carbaryl	12	hours	water	21	3	4	3	4	https://www.sciencedirect.com/science/article/pii/S0166445X1830345X	
Poecilus	insect	Cd	90	days	food	20	1	0.0351	0.0308	0.0392	http://dx.doi.org/10.1371/journal.pone.0108740	
Radix	aquatic invertebrate	BDE99 BDE47	9	days	sediment	10	1	100	2	2276	https://tel.archives-ouvertes.fr/tel-02612792	biotransformation, in mixture with PCB153
Radix	aquatic invertebrate	cypermethrine	24	hours	water	16	2	902	766	1040	http://dx.doi.org/10.1021/acs.est.0c00189	
Radix	aquatic invertebrate	PCB153	9	days	sediment	10	1	111	4	1781	https://tel.archives-ouvertes.fr/tel-02612792	in mixture with BDE99
Snail	terrestrial invertebrate	Mn	28	days	sediment	32	5	0.2124	0.1788	0.2477		3 tested concentrations
Solea	fish	PCB105	90	days	food	36	3	0.4912	0.4	0.6163	https://doi.org/10.1016/j.seares.2010.05.005	in mixture
Solea	fish	PCB153	90	days	food	34	3	0.4993	0.3921	0.6722	https://doi.org/10.1016/j.seares.2010.05.005	in mixture
Tubifex	aquatic worm	cypermethrine	24	hours	water	16	2	77492	5384	1784701	http://dx.doi.org/10.1021/acs.est.0c00189	
Apostichopus	marine invertebrate	azithromycin	28	days	water	19	1	81	74	88	https://doi.org/10.1021/acs.est.0c04421	
Apostichopus	marine invertebrate	clarithromycin	28	days	water	19	1	92	80	104	https://doi.org/10.1021/acs.est.0c04421	
Apostichopus	marine invertebrate	enrofloxacin	28	days	water	19	1	75	69	82	https://doi.org/10.1021/acs.est.0c04421	
Apostichopus	marine invertebrate	ofloxacin	28	days	water	19	1	39	34	45	https://doi.org/10.1021/acs.est.0c04421	
Apostichopus	marine invertebrate	sulfadiazine	28	days	water	18	1	6	5	7	https://doi.org/10.1021/acs.est.0c04421	
Apostichopus	marine invertebrate	trimethoprim	28	days	water	18	1	51	46	57	https://doi.org/10.1021/acs.est.0c04421	
Brachydanio	fish	atrazine	1	days	water	37	1	1	1	2	https://www.sciencedirect.com/science/article/pii/014765139090004O	
Cloeon	insect	imidacloprid	2.02	days	water	33	3	6174	147	119522	https://www.sciencedirect.com/science/article/pii/S0166445X21000965	biotransformation
Cyprinodon	fish	naphtalene	36	days	water	14	1	7	4	4231	https://setac.onlinelibrary.wiley.com/doi/abs/10.1897/03-173	in mixture
Cyprinodon	fish	phenantrene	36	days	water	13	1	526	17	378268	https://setac.onlinelibrary.wiley.com/doi/abs/10.1897/03-173	in mixture
Cyprinodon	fish	pyrene	36	days	water	14	1	28	19	3636	https://setac.onlinelibrary.wiley.com/doi/abs/10.1897/03-173	in mixture
Eisenia	aquatic worm	C14	21	days	sediment	6	1	20	4	827	http://dx.doi.org/10.1007/s10646-017-1769-4	
Gallus	vertebrate	alpha-HBCD	21	days	sediment	10	1	0.0212	0.001	0.1529	https://doi.org/10.1007/s11356-011-0573-6	liver
Gallus	vertebrate	gamma-HBCD	21	days	sediment	10	1	0.0171	0.0148	0.019	https://doi.org/10.1007/s11356-011-0573-6	liver
Gallus	vertebrate	HBCD	21	days	sediment	10	1	2	0	161	https://doi.org/10.1007/s11356-011-0573-6	liver, biotransformation
Gallus	vertebrate	PFOS	102	days	food	25	1	743	580	1014	https://www.sciencedirect.com/science/article/pii/S037842741401474X	
Gammarus	aquatic invertebrate	azoxistrobine	1	days	water	38	2	4524	1662	8993041	https://doi.org/10.1021/acs.est.7b03088	biotransformation, in mixture
Gammarus	aquatic invertebrate	epoxiconazole	1	days	water	34	3	85	74	91	https://doi.org/10.1021/acs.est.6b01301	biotransformation
Gammarus	aquatic invertebrate	imidachloprid	168	hours	water	16	1	9	8	11	https://doi.org/10.1021/acs.est.0c07839	H2 site
Gammarus	aquatic invertebrate	imidachloprid	168	hours	water	16	1	9	7	11	https://doi.org/10.1021/acs.est.0c07839	H6 site

Go to next page

Table S2. *(continued).*

genus	category	substance	tacc	tunit	exposure	ndata	nrep	bioacc	bioaccQinf	bioaccQsup	DOI	comment
Gammarus	aquatic invertebrate	imidacloprid	14	days	water	13	10	6	5	7	https://doi.org/10.1371/journal.pone.0062472	total radioactivity
Gammarus	aquatic invertebrate	imidacloprid	2.02	days	water	33	3	61	44	97	https://www.sciencedirect.com/science/article/pii/S0166445X21000965	biotransformation
Gammarus	aquatic invertebrate	prochloraz	1	days	water	30	2	111	104	118	https://doi.org/10.1021/acs.est.7b03088	in mixture
Gammarus	aquatic invertebrate	propiconazole	24	hours	water	7	3	14	12	17	https://doi.org/10.1007/s10646-012-0917-0	total radioactivity
Gammarus	aquatic invertebrate	pyrene	7	days	water	28	4	157166	1719	9280570	https://www.sciencedirect.com/science/article/pii/S0141113615300113	biotransformation, total radioactivity
Lumbriculus	aquatic worm	atrazine	240	days	sediment	16	3	0.031	0.0219	0.0498	https://www.sciencedirect.com/science/article/pii/S0147651308000201	2 tested concentrations, use of radiotracers
Lumbriculus	aquatic worm	chlorpyrifos	240	days	sediment	18	3	2	1	15	https://www.sciencedirect.com/science/article/pii/S0147651308000201	2 tested concentrations, use of radiotracers
Metaphire	aquatic worm	C14	21	days	sediment	6	1	18	13	29	http://dx.doi.org/10.1007/s10646-017-1769-4	
Mytilus	aquatic invertebrate	8-2-diPAP	72	hours	water	18	1	838	575	1147	https://doi.org/10.1002/etc.5060	
Oryzias	fish	diSPAP	10	days	water	10	1	40	0	14261	https://doi.org/10.1021/es404867w	biotransformation
Potamocorbula	marine invertebrate	Cd	72	hours	water	12	7	0	0	3	https://doi.org/10.1021/acs.est.0c06644	7 tested concentrations, in mixture, use of radio-tracers
Salmo	fish	1,2-DCB	119	days	water	5	2	0.2591	0.2075	0.3035	https://doi.org/10.1021/es00111a009	2 tested concentrations, in mixture
Salmo	fish	1,2,3-TCB	119	days	water	5	2	1	1	1	https://doi.org/10.1021/es00111a009	2 tested concentrations, in mixture
Salmo	fish	1,2,3,4-TeCB	119	days	water	5	2	5	3	7	https://doi.org/10.1021/es00111a009	2 tested concentrations, in mixture
Salmo	fish	1,2,4-TCB	199	days	water	4	2	1	0	3	https://doi.org/10.1021/es00111a009	2 tested concentrations, in mixture
Salmo	fish	1,2,4,5-TeCB	119	days	water	5	2	6	4	9	https://doi.org/10.1021/es00111a009	2 tested concentrations, in mixture
Salmo	fish	1,3-DCB	119	days	water	5	2	0.4415	0.385	0.4903	https://doi.org/10.1021/es00111a009	2 tested concentrations, in mixture
Salmo	fish	1,3,5-TCB	119	days	water	5	2	2	2	2	https://doi.org/10.1021/es00111a009	2 tested concentrations, in mixture
Salmo	fish	1,4-DCB	119	days	water	5	2	0.3857	0.2675	0.4896	https://doi.org/10.1021/es00111a009	2 tested concentrations, in mixture
Salmo	fish	hCB	119	days	water	5	2	116	5	2624	https://doi.org/10.1021/es00111a009	2 tested concentrations, in mixture
Salmo	fish	HCBD	119	days	water	5	2	6	4	8	https://doi.org/10.1021/es00111a009	2 tested concentrations, in mixture
Salmo	fish	HCE	119	days	water	8	2	0.713	0.564	0.8628	https://doi.org/10.1021/es00111a009	2 tested concentrations, in mixture
Salmo	fish	QCB	119	days	water	5	2	17	0	135	https://doi.org/10.1021/es00111a009	2 tested concentrations, in mixture
Spirostomum	Heterotrichea	fluoxetine	6	days	water	14	2	6870	525	461623	https://www.mdpi.com/1420-3049/25/7/1476	3 tested concentrations
Spirostomum	Heterotrichea	mianserin	6	days	water	14	2	3142	1991	4244	https://www.mdpi.com/1420-3049/25/7/1476	3 tested concentrations
Spirostomum	Heterotrichea	paroxetine	6	days	water	14	2	189	1	553	https://www.mdpi.com/1420-3049/25/7/1476	3 tested concentrations
Spirostomum	Heterotrichea	sertraline	6	days	water	14	2	82875	6030	8203084	https://www.mdpi.com/1420-3049/25/7/1476	3 tested concentrations
Tenebrio	insect	Ag2S-NPs-226mg	21	days	sediment	12	1	0.0938	0.0626	0.1231	https://www.sciencedirect.com/science/article/pii/S0048969721011384	in mixture
Tenebrio	insect	Ag2S-NPs-22mg	21	days	sediment	12	1	0.0157	0.0106	0.0204	https://www.sciencedirect.com/science/article/pii/S0048969721011384	in mixture
Tenebrio	insect	Ag2S-NPs	21	days	food	12	1	0.0881	0.0454	0.1273	https://www.sciencedirect.com/science/article/pii/S0048969721011384	in mixture
Tenebrio	insect	AgNO3	21	days	sediment	12	1	0.2597	0.0993	0.396	https://www.sciencedirect.com/science/article/pii/S0048969721011384	in mixture
Tenebrio	insect	AgNO3	21	days	food	12	1	0	0	5	https://www.sciencedirect.com/science/article/pii/S0048969721011384	in mixture
Tenebrio	insect	AgNPs3-8nm	21	days	sediment	12	1	0.2073	0.1256	0.2767	https://www.sciencedirect.com/science/article/pii/S0048969721011384	in mixture
Tenebrio	insect	AgNPs3-8nm	21	days	food	12	1	0	0	11	https://www.sciencedirect.com/science/article/pii/S0048969721011384	in mixture
Tenebrio	insect	AgNPs50nm	21	days	sediment	11	1	0	0	8	https://www.sciencedirect.com/science/article/pii/S0048969721011384	in mixture
Tenebrio	insect	AgNPs50nm	21	days	food	12	1	0.0757	0.0386	0.1099	https://www.sciencedirect.com/science/article/pii/S0048969721011384	in mixture
Tenebrio	insect	AgNPs60nm	21	days	sediment	11	1	0.2472	0.1304	0.3489	https://www.sciencedirect.com/science/article/pii/S0048969721011384	in mixture
Tenebrio	insect	AgNPs60nm	21	days	food	12	1	0.1329	0	0.2429	https://www.sciencedirect.com/science/article/pii/S0048969721011384	in mixture
Tigriopus	marine invertebrate	HBCD	96	hours	water	10	1	92978	79361	112386	https://www.sciencedirect.com/science/article/pii/S0045653516313728	commercial mixture
Danio	fish	emodin	48	hours	water	9	2	123	100	143	http://dx.doi.org/10.1007/s10646-017-1769-4	2 tested concentrations
Enchytraeus	terrestrial worm	AgNP-Cit	10	days	water	5	6	18	0	250409	https://setac.onlinelibrary.wiley.com/doi/abs/10.1002/etc.3123	6 tested concentrations, in mixture
Enchytraeus	terrestrial worm	AgNP-PVP	10	days	water	5	4	56	0	523281	https://setac.onlinelibrary.wiley.com/doi/abs/10.1002/etc.3123	4 tested concentrations, in mixture
Metaphire	aquatic worm	6-2-diPAP	21	days	sediment	10	1	323	1	63154241	https://www.sciencedirect.com/science/article/pii/S0160412021000763	biotransformation
Anax	aquatic invertebrate	chlorpyrifos	2.313	days	water	49	5	113	82	181	https://setac.onlinelibrary.wiley.com/doi/abs/10.1002/etc.273	total radioactivity
Asellus	aquatic invertebrate	chlorpyrifos	2.048	days	water	48	5	3273	2570	4558	https://setac.onlinelibrary.wiley.com/doi/abs/10.1002/etc.273	total radioactivity
Chaoborus	aquatic invertebrate	chlorpyrifos	1.958	days	water	97	5	2859	2128	4508	https://setac.onlinelibrary.wiley.com/doi/abs/10.1002/etc.273	total radioactivity
Cloeon	aquatic invertebrate	chlorpyrifos	2.031	days	water	98	10	1825	1464	2376	https://setac.onlinelibrary.wiley.com/doi/abs/10.1002/etc.273	total radioactivity
Culex	aquatic invertebrate	chlorpyrifos	2.396	days	water	49	5	270910	5565	21437990	https://setac.onlinelibrary.wiley.com/doi/abs/10.1002/etc.273	total radioactivity
Daphnia	aquatic invertebrate	chlorpyrifos	2.042	days	water	47	5	543	461	652	https://setac.onlinelibrary.wiley.com/doi/abs/10.1002/etc.273	total radioactivity
Gammarus	aquatic invertebrate	chlorpyrifos	2.041	days	water	48	5	3886	2159	1072296	https://setac.onlinelibrary.wiley.com/doi/abs/10.1002/etc.273	total radioactivity
Gammarus	aquatic invertebrate	chlorpyrifos	2.048	days	water	49	5	2459	1931	3322	https://setac.onlinelibrary.wiley.com/doi/abs/10.1002/etc.273	total radioactivity
Molanna	aquatic invertebrate	chlorpyrifos	2.021	days	water	50	5	7294	4917	17538	https://setac.onlinelibrary.wiley.com/doi/abs/10.1002/etc.273	total radioactivity
Neocaridina	aquatic invertebrate	chlorpyrifos	2.021	days	water	49	5	1982	1349	3880	https://setac.onlinelibrary.wiley.com/doi/abs/10.1002/etc.273	total radioactivity
Notonecta	aquatic invertebrate	chlorpyrifos	2.052	days	water	50	5	443	362	571	https://setac.onlinelibrary.wiley.com/doi/abs/10.1002/etc.273	total radioactivity

Go to next page

Table S2. *(continued).*

genus	category	substance	tacc	tunit	exposure	ndata	nrep	bioacc	bioaccQinf	bioaccQsup	DOI	comment
Parapoynx	aquatic invertebrate	chlorpyrifos	2.063	days	water	50	5	2110	1534	3624	https://setac.onlinelibrary.wiley.com/doi/abs/10.1002/etc.273	total radioactivity
Plea	aquatic invertebrate	chlorpyrifos	2.073	days	water	45	5	716	474	2120	https://setac.onlinelibrary.wiley.com/doi/abs/10.1002/etc.273	total radioactivity
Procambarus	aquatic invertebrate	chlorpyrifos	1.999	days	water	47	5	7632	931	9526021	https://setac.onlinelibrary.wiley.com/doi/abs/10.1002/etc.273	total radioactivity
Procambarus	aquatic invertebrate	chlorpyrifos	2.021	days	water	48	5	5874	268	1529247	https://setac.onlinelibrary.wiley.com/doi/abs/10.1002/etc.273	total radioactivity
Rantra	aquatic invertebrate	chlorpyrifos	1.977	days	water	49	5	925	288	1937369	https://setac.onlinelibrary.wiley.com/doi/abs/10.1002/etc.273	total radioactivity
Sialis	aquatic invertebrate	chlorpyrifos	2.094	days	water	39	5	335606	6355	16281251	https://setac.onlinelibrary.wiley.com/doi/abs/10.1002/etc.273	total radioactivity
Daphnia	aquatic invertebrate	anthracene	24	hours	food	26	3	0.0114	0.0104	0.0124	https://doi.org/10.1021/acs.est.0c06970	body without gut, in mixture, use of radiotracers
Daphnia	aquatic invertebrate	anthracene	24	hours	water	23	3	635	558	708	https://doi.org/10.1021/acs.est.0c06970	body without gut, in mixture, use of radiotracers
Daphnia	aquatic invertebrate	anthracene	24	hours	food	25	3	0.0412	0.0357	0.0462	https://doi.org/10.1021/acs.est.0c06970	gut, in mixture, use of radiotracers
Daphnia	aquatic invertebrate	anthracene	24	hours	water	24	3	2237	1984	2485	https://doi.org/10.1021/acs.est.0c06970	gut, in mixture, use of radiotracers
Daphnia	aquatic invertebrate	fluoranthene	24	hours	food	26	3	0.0069	0.0062	0.0077	https://doi.org/10.1021/acs.est.0c06970	body without gut, in mixture, use of radiotracers
Daphnia	aquatic invertebrate	fluoranthene	24	hours	water	22	3	248	212	283	https://doi.org/10.1021/acs.est.0c06970	body without gut, in mixture, use of radiotracers
Daphnia	aquatic invertebrate	fluoranthene	24	hours	food	24	3	0.0443	0.0407	0.0478	https://doi.org/10.1021/acs.est.0c06970	gut, in mixture, use of radiotracers
Daphnia	aquatic invertebrate	fluoranthene	24	hours	water	19	3	1215	1050	1376	https://doi.org/10.1021/acs.est.0c06970	gut, in mixture, use of radiotracers
Daphnia	aquatic invertebrate	phenanthrene	24	hours	food	27	3	0.0097	0.009	0.0104	https://doi.org/10.1021/acs.est.0c06970	body without gut, in mixture, use of radiotracers
Daphnia	aquatic invertebrate	phenanthrene	24	hours	water	21	3	438	397	478	https://doi.org/10.1021/acs.est.0c06970	body without gut, in mixture, use of radiotracers
Daphnia	aquatic invertebrate	phenanthrene	24	hours	food	24	3	0.0349	0.0265	0.039	https://doi.org/10.1021/acs.est.0c06970	gut, in mixture, use of radiotracers
Daphnia	aquatic invertebrate	phenanthrene	24	hours	water	25	3	993	934	1054	https://doi.org/10.1021/acs.est.0c06970	gut, in mixture, use of radiotracers
Daphnia	aquatic invertebrate	pyrene	24	hours	food	25	3	0.012	0.011	0.013	https://doi.org/10.1021/acs.est.0c06970	body without gut, in mixture, use of radiotracers
Daphnia	aquatic invertebrate	pyrene	24	hours	water	25	3	520	453	587	https://doi.org/10.1021/acs.est.0c06970	body without gut, in mixture, use of radiotracers
Daphnia	aquatic invertebrate	pyrene	24	hours	food	25	3	0.0777	0.071	0.0822	https://doi.org/10.1021/acs.est.0c06970	gut, in mixture, use of radiotracers
Daphnia	aquatic invertebrate	pyrene	24	hours	water	18	3	2163	1964	2355	https://doi.org/10.1021/acs.est.0c06970	gut water, in mixture, use of radiotracers
Enchytraeus	terrestrial worm	phenanthrene	14	days	sediment	16	1	228	209	246	https://doi.org/10.1021/acs.est.0c06182	10.1 ◦ C
Enchytraeus	terrestrial worm	phenanthrene	14	days	sediment	16	1	242	207	277	https://doi.org/10.1021/acs.est.0c06182	14.3 ◦ C
Enchytraeus	terrestrial worm	phenanthrene	14	days	sediment	16	1	284	250	317	https://doi.org/10.1021/acs.est.0c06182	20.3 ◦ C
Enchytraeus	terrestrial worm	phenanthrene	14	days	sediment	16	1	327	275	368	https://doi.org/10.1021/acs.est.0c06182	24.7 ◦ C
Enchytraeus	terrestrial worm	phenanthrene	14	days	sediment	16	1	179	153	209	https://doi.org/10.1021/acs.est.0c06182	5.3 ◦ C
Enchytraeus	terrestrial worm	phenanthrene	14	days	sediment	16	1	265	246	285	https://doi.org/10.1021/acs.est.0c06182	daily temperature variation
Hyalella	aquatic invertebrate	diclofenac	1	days	water	30	2	490	1	496477	https://doi.org/10.1021/acs.est.0c07887	Cext constantbiotransformation
Hyalella	aquatic invertebrate	diclofenac	1	days	water	40	2	848	2	828853	https://doi.org/10.1021/acs.est.0c07887	Cext variablebiotransformation
Perca	fish	SAmPAP	45	days	food	13	1	1	0	16	https://doi.org/10.1021/acs.est.6b05598	liver, biotransformation
Perca	fish	SAmPAP	45	days	food	13	1	0	0	2	https://doi.org/10.1021/acs.est.6b05598	muscle, biotransformation
Perca	fish	SAmPAP	45	days	food	13	1	1	0	33	https://doi.org/10.1021/acs.est.6b05598	serum, biotransformation
Daphnia	aquatic invertebrate	uranium	2	days	water	6	4	5322	4551	6105	https://www.sciencedirect.com/science/article/pii/S0166445X21000953	4 tested concentrations, use of radiotracers
Eisenia	terrestrial worm	bifenthrin	504	hours	sediment	14	1	0.126	0.1141	0.1388	https://setac.onlinelibrary.wiley.com/doi/abs/10.1002/etc.4094	
Eisenia	terrestrial worm	capture-LFR	504	hours	sediment	14	1	0.1568	0.1397	0.1763	https://setac.onlinelibrary.wiley.com/doi/abs/10.1002/etc.4094	
Eisenia	terrestrial worm	nano-A	504	hours	sediment	14	1	0.1773	0.1663	0.1879	https://setac.onlinelibrary.wiley.com/doi/abs/10.1002/etc.4094	
Eisenia	terrestrial worm	nano-B	504	hours	sediment	14	1	0.2004	0.1895	0.2112	https://setac.onlinelibrary.wiley.com/doi/abs/10.1002/etc.4094	
Enchytraeus	terrestrial worm	AgNO3	10	days	water	10	2	2993	0	219390	https://setac.onlinelibrary.wiley.com/doi/abs/10.1002/etc.3123	4 tested concentrations
Gammarus	aquatic invertebrate	Cd	7	days	water	18	3	25808	19020	35682	https://www.sciencedirect.com/science/article/pii/S0160412021002506	caeca, use of radiotracers
Gammarus	aquatic invertebrate	Cd	7	days	water	18	3	1568	1131	2235	https://www.sciencedirect.com/science/article/pii/S0160412021002506	cephalons, use of radiotracers

Go to next page

Table S2. *(continued).*

genus	category	substance	tacc	tunit	exposure	ndata	nrep	bioacc	bioaccQinf	bioaccQsup	DOI	comment
Gammarus	aquatic invertebrate	Cd	7	days	water	18	3	3792	2774	5067	https://www.sciencedirect.com/science/article/pii/S0160412021002506	intestins, use of radiotracers
Gammarus	aquatic invertebrate	Cd	7	days	water	18	3	5140	3681	7805	https://www.sciencedirect.com/science/article/pii/S0160412021002506	tissues, use of radiotracers
Gammarus	aquatic invertebrate	Hg	7	days	water	18	3	7832	4459	12687	https://www.sciencedirect.com/science/article/pii/S0160412021002506	caeca, use of radiotracers
Gammarus	aquatic invertebrate	Hg	7	days	water	21	3	41749	8893	2099539	https://www.sciencedirect.com/science/article/pii/S0160412021002506	cephalons, use of radiotracers
Gammarus	aquatic invertebrate	Hg	7	days	water	21	3	7449	5576	10231	https://www.sciencedirect.com/science/article/pii/S0160412021002506	intestins, use of radiotracers
Gammarus	aquatic invertebrate	Hg	7	days	water	21	3	11968	7195	25250	https://www.sciencedirect.com/science/article/pii/S0160412021002506	tissues, use of radiotracers
